# Biomechanical conditions of subtalar joint arthrodesis with calcaneal locking nail: A probabilistic numerical study

**DOI:** 10.1371/journal.pone.0314034

**Published:** 2024-11-20

**Authors:** Timon Pahl, Albrecht Radtke, Joana F. Büttner, Thomas Mittlmeier, Philipp Weißgraeber

**Affiliations:** 1 Faculty of Mechanical Engineering and Marine Technology, Chair of Lightweight Design, University of Rostock, Rostock, Germany; 2 Dept. of Trauma, Hand and Reconstructive Surgery, Rostock University Medical Center, Rostock, Germany; University of Perugia: Universita degli Studi di Perugia, ITALY

## Abstract

**Introduction:**

Subtalar joint arthrodesis is primarily indicated for advanced osteoarthritis, hindfoot deformity, and/or instability. During the first 6-10 weeks after surgery, there is an intermediary structurally weaker state before complete bony fusion of the calcaneus and talus occurs. Loading of the foot can lead to mechanical stresses and relative movements in the former joint gap, which can impede the fusion process. The objective of this study was to examine the mechanical healing conditions for a subtalar arthrodesis with a calcaneal locking nail.

**Methods:**

A probabilistic finite element model of the subtalar joint with a calcaneal locking nail was created to represent the foot post-surgery that accounts for the uncertainty of the material properties. The model differentiates between cortical and cancellous bone and includes non-linear contact definitions in the subtalar joint. Multiple loading scenarios, including hindfoot inversion/eversion, were simulated to determine bone and implant stresses. Utilizing local articular coordinate systems, a displacement analysis was established to separate normal and tangential components and account for their separate effects. The loading of the locking nail was assessed through section moments.

**Results:**

Under inversion/eversion loading, the area near the locking screws and upper end of the nail experienced the highest stresses. The maximum stresses in cortical and cancellous bone were 112±8.3 MPa and 2.1±0.2 MPa, respectively. The comparison of the von Mises and maximum principal stresses for the bones showed a load case dependency with strong effect on tensile loading states. The proposed method for the analysis of relative displacement in the local articular coordinate systems showed joint regions exhibiting normal and tangential movements that changed with the considered loading states. It was found that tangential displacements of up to 0.19 mm are related to the torsional loading of the calcaneal locking nail, which is connected to the corresponding torsional stiffness of the implant and its fixation in the calcaneus and talus. Normal displacements in the joint gap of up to -0.18 mm can be shown to be governed by the bending moments acting on the calcaneal locking nail, which are linked to the nail’s bending stiffness. The ratio of tangential and normal displacement in the critical inversion configuration was determined to be -1.1.

**Conclusions:**

Inversion and eversion loads can lead to significant mechanical loading of the bones and to bending and torsional loading of the locking nail. The bending leads to normal displacements in the articular gap. Torsions can lead to significant tangential displacements that have been shown to promote non-union instead of bony fusion.

## Introduction

**Fig 1:**
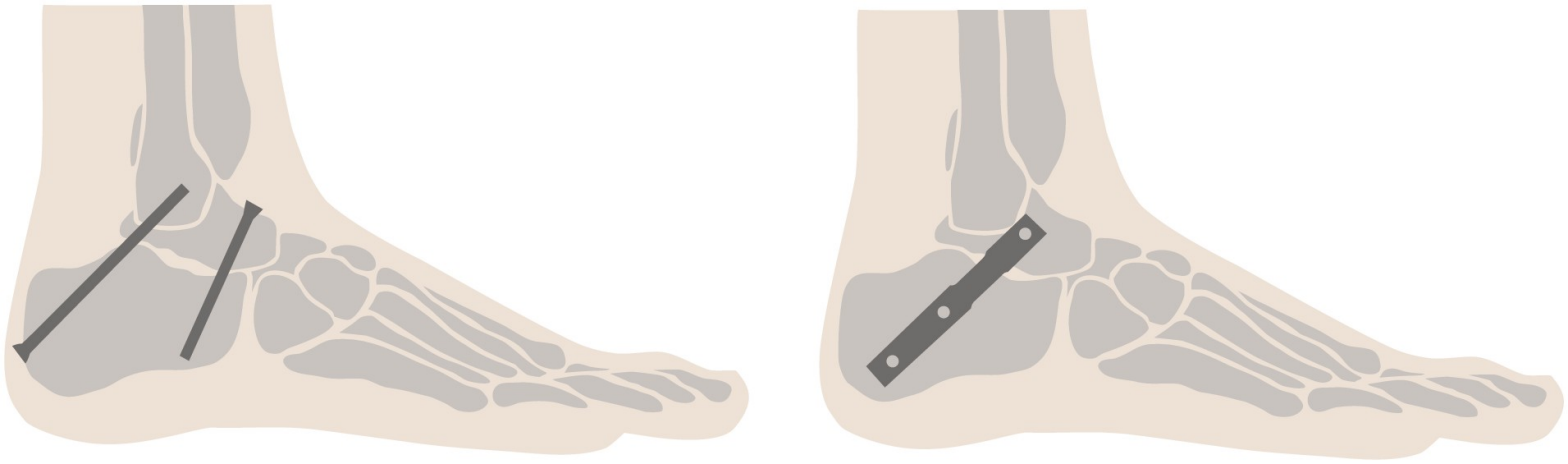
Examples of surgical treatment methods for arthrodesis. Principle sketches for surgical treatment methods of subtalar arthrodesis from lateral view: screw technique employing two non-parallel screws (left), interlocking nail technique (right).

Osteoarthritis, joint malalignment, and ankle instability can significantly limit mobility and cause severe pain, reducing quality of life. The worldwide prevalence of osteoarthritis is increasing [1]. Arthrodesis has the potential to enhance quality of life and alleviate pain by stiffening affected joints through the fusion of bones. In cases of subtalar joint malalignment accompanied by a realignment procedure, subtalar arthrodesis specifically fuses the calcaneus and talus [2]. A significant indication for subtalar joint arthrodesis is posttraumatic osteoarthritis resulting from an intraarticular calcaneal fracture. A range of techniques can be employed to achieve this, with the use of one to three separate screws representing the most prevalent approach (refer to Fig 1) [3]. Saß et al. [4] reported that hollow intramedullary nails are becoming an increasingly important therapeutic option in this context. These nails are commonly used due to their high implant stiffness including the application of angular stable fixation and the advantages of soft-tissue sparing minimally invasive procedures.

Bone healing depends on several factors, such as blood supply, hormones, growth factors, and mechanical healing conditions [5–7]. According to literature the biomechanical environment represents a central factor [8, 9]. In the absence of bony healing, a non-union may occur with an incidence rate of 3–20% necessitating surgical revision in symptomatic patients [10, 11]. It is to be noted that the effect of shear displacements on healing is a subject of debate within the literature. According to several studies, optimized compressive stimuli promote healing, while torsion and shear loads do not [9, 12, 13]. It is also worth noting that the identification of tangential displacement using conventional imaging techniques is challenging. In order to understand the biomechanical healing conditions in arthrodesis, it is necessary to study the interaction between the stiff implants and the bone in more detail. To this end we provide a comprehensive probabilistic numerical study.

Therefore, a finite element (FE) model simplifying the bony anatomy of the hindfoot immediately after surgery was developed to investigate the healing conditions of subtalar arthrodesis with an intramedullary triplicate interlocking nail. The present study is primarily concerned with the initial mechanical healing conditions, as evidence from previous research indicates that these may exert a considerable influence on the eventual clinical outcome [8, 9, 14]. To ascertain the comparability of real and artificial bones, the study was conducted using both types of specimens. To investigate different loading conditions for a single-leg stance, the model was progressively tilted to the maximum physiological inversion (*φ* = -20°) and eversion (*φ* = 10°) configuration [15]. The uncertainty of the material properties used as model input is taken into account in the probabilistic finite element analysis study. The aim of this study is to achieve a better understanding of the healing conditions, to provide insights for following clinical studies and to support evidence-based clinical decision-making in orthopedic surgery.

## Materials and methods

The main methodology of this study involved developing a computer-based model of subtalar arthrodesis, incorporating the talus, calcaneus, and the hollow intramedullary nail in order to study the biomechanical conditions within the stiffened joint. The methodology is based on a comprehensive analysis of bone geometry, material properties, boundary and load conditions, and the identification of indicators of the biomechanical conditions under loading. These indicators include the stresses in the cortical and cancellous bones, the stresses in the implant and the relative displacements in the articular gap. Within this analysis, a probabilistic methodology was employed to accommodate potential variabilities in material properties and assess their effect on the study results.

### Biphasic three-dimensional geometric model establishment

To obtain the external geometry of the bones, we used a three-dimensional computer aided design (CAD) model of the right foot [16, 17] obtained from computed tomography (CT) data of a 29-year-old male weighing 65 kg and made minor adjustments to represent the condition of a subtalar arthrodesis. To account for the heterogeneous bone structure at the macroscale of the CAD model, we implemented one phase each for cortical and cancellous bone. Additionally, we identified the posterior part of the tuber calcanei and the joint surfaces that articulate with the talus as areas with increased wall thickness, following Sabry et al. [18], assuming that the average wall thickness of the cortical bone in the talus is the same as in the calcaneus (2.5±0.4 mm). Cortical wall thickness in the talus is significantly higher at the posterior calcaneal articular surface along with the lateral and medial articular surfaces up to the ankle joint and the talar cervical circumference [19]. For the areas that showed an increase in wall thickness, we combined the mean wall thickness value with the standard deviation derived from Sabry et al.’s study [18]. In areas showing reduced wall thickness, we subtracted the standard deviation from the mean wall thickness. Autodesk’s Fusion 360 v2.0 software was used to implement the biphasic model of the talus and calcaneus with varying wall thickness using mesh bodies and Boolean operations.

**Fig 2:**
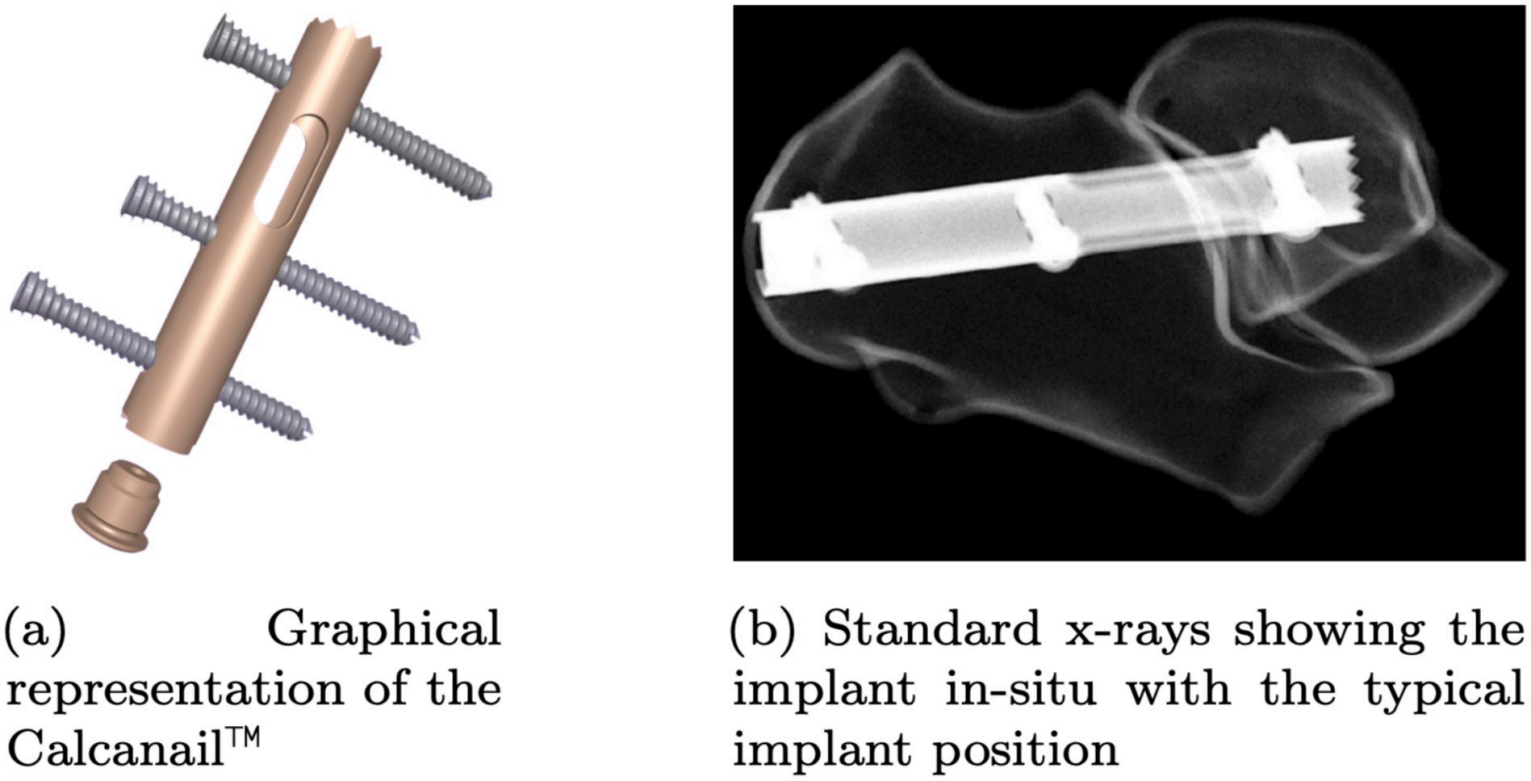
Calcanail™ from FH Ortho SAS. Detailed view of the Calcanail™ implant used in orthopedic surgery.

In the actual surgical procedures, the cartilage on the joint surfaces is removed, and any sclerotic bone is debrided. To achieve a realistic arrangement of the bones in the model, the distance between the talus and calcaneus at the articular surfaces was set to approximately 1 mm. In the CAD model the implant was placed according to the surgical technique recommended by the manufacturer of an exemplaric calcaneal locking nail (Calcanail™ FH Ortho SAS (Heimsbrunn, France), which is a intramedullary interlocking nail (see Fig 2)). The Calcanail™ is a hollow cylindrical base body with one oblong hole and three locking screw holes along its length, arranged in parallel and evenly distributed, which is made of an titanium alloy. The implant is 75 mm long, 12 mm in diameter, and has a wall thickness of 2 mm. The tooth profile and the cap were simplified to idealized versions. The locking screws with a nominal diameter of 5 mm were simplified to cylinders with their core diameter size 3.7 mm.

The CAD model was imported into Simulia Abaqus 2021 software for Finite Element Analysis (FEA). FEA is a numerical technique that discretizes a physical domain into a mesh of finite elements. It solves the equilibrium equations, typically derived from the principles of mechanics and elasticity, for each element using methods such as the stiffness matrix and nodal displacement vectors. In biomechanics, FEA models biological tissues and implants by solving the governing equations of motion and material deformation, often incorporating nonlinear material properties and contact interactions. This enables precise simulations of stress, strain, and deformation under various loading conditions, facilitating the optimization of medical device design and the prediction of physiological responses [20]. The model’s structures were discretized into tetrahedral elements using the quadratic attachment function (C3D10). A convergence study was conducted prior to the actual analysis to validate the selected model discretization, ensuring a appropriate model accuracy and computational efficiency. A characteristic element size of 1.44 mm was identified as the optimal choice, amounting to a total of 427 538 elements for the entire model.

### Boundary and load conditions

**Fig 3:**
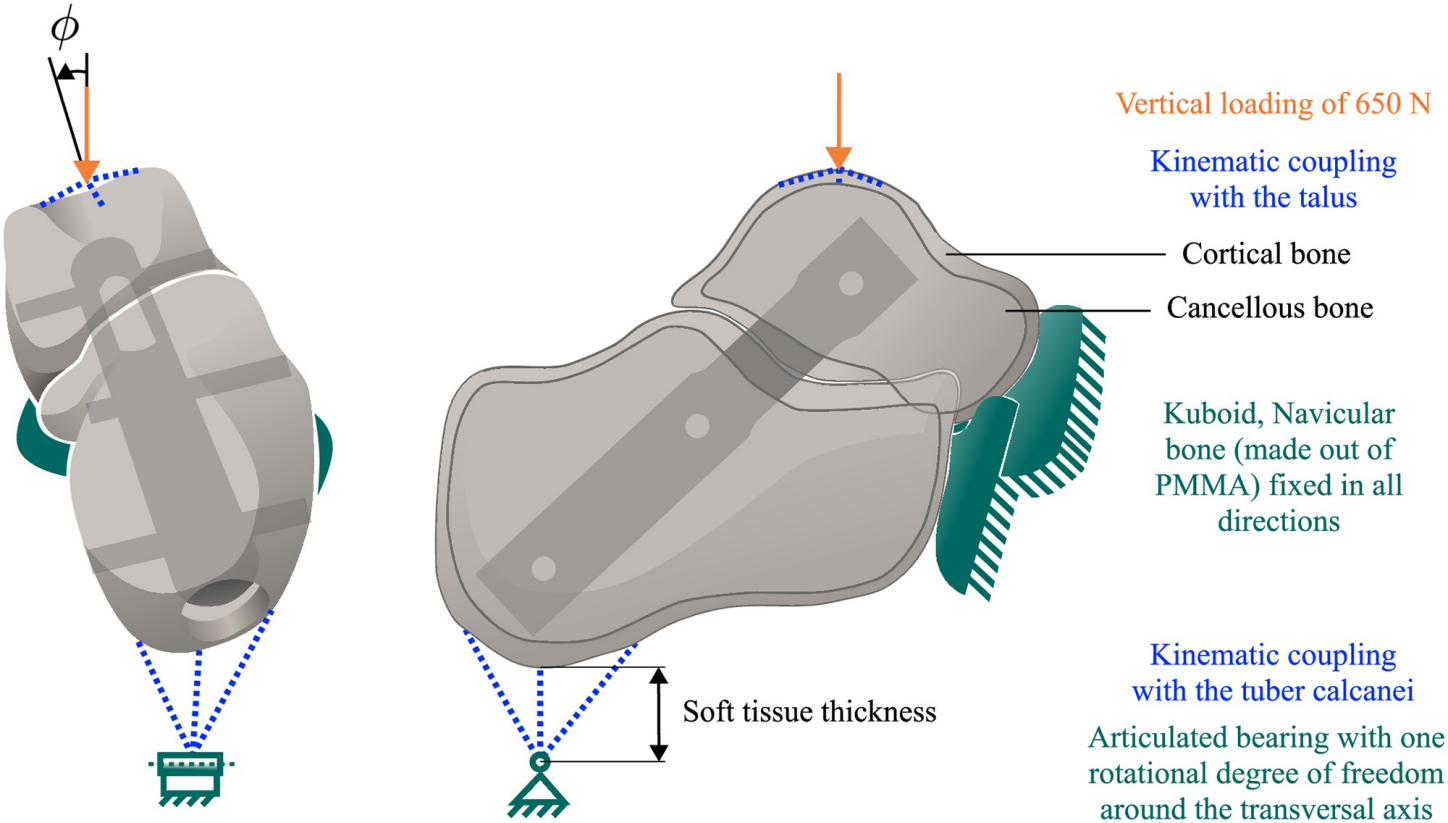
Treading loading scenarios for the bi-phasic bone-model. Treading loading scenarios depending on *φ* (-20° ≤*φ*≤ 10°) with load application and boundary conditions of the bi-phasic bone-model from posterior (left) and lateral view (right) defined in the numerical study according to the experimental study by [21].

Tilted configurations considered simplified single-leg stance with various angles *φ* of eversion and inversion of the foot up to a the physiological maximum of 10° and -20° [15] to simulate treading with a rotated ankle (*φ* = 0°, neutral position) (see Fig 3). In the parametric variations, the angle *φ* was incremented in intervals of 5°. A vertical force of 650 N simulating the body weight of the person that the bone model was derived from [17] was applied to a reference point kinematically coupled with the proximal articular surface of the talus. A second reference point with only one rotational degree of freedom about the transverse axis was kinematically coupled with the tuber calcanei. It was placed 17 mm below the tuber calcanei to account for the thickness of the soft tissue according to the model of Franciosa et al. [17]. To simulate a simplified forefoot’s support at the anterior parts of the talus and calcaneus the articular surfaces of the cuboid and navicular bones (made out of Poly(methyl methacrylate) (PMMA)) were modelled with a fixed boundary condition similar to the studies of [21] (Fig 3).

The screws of the hollow intramedullary nail, the inner surface of the cortical bone, and the outer surface of the cancellous bone were modeled together. Following the methodology of Ni et al. [22] a normal and sliding contact with friction (*μ* = 0.3) was defined between talus, calcaneus and the implant. This contact definition introduces non-linearity to the boundary value problem, which requires the use of a non-linear solver in the finite element analysis (FEA) as detailed below.

### Material properties

The material parameters used in this study for real bone, artificial bone, titanium implants, and PMMA bone cement are presented in Table 1. For the considered homogeneous materials we defined linear-elastic, isotropic material behavior. The material properties of the artificial bones were derived from the material characteristics of Sawbones, which is a product of Sawbones Europe AB (Malmö, Sweden). Sawbones products are designed to imitate the mechanical behavior of real bones and have been developed for experimental studies and training in surgical procedures [23].

**Table 1:** Material parameters for cortical bone, cancellous bone, and titanium with their mean values and standard deviations (SD).

Material	Young’s modulus (mean (SD)) [MPa]	Poisson’s ratio [-]	References
Cortical bone	17,000 (3,400)	0.3	[22, 37, 38]
Cancellous bone	100 (20)	0.3	[31, 39]
Artificial cortical bone	16,350 (1,635)	0.3	[23]
Artificial cancellous bone	155 (16)	0.3	[23]
Titanium alloy	110,000 (4,400)	0.3	[22, 29–31]
PMMA bone cement	3,000 (300)	0.4	[40]

In reality, material properties vary, particularly in real bone due to biological variables such as genetics, age, sex, level of activity, and possible pathological degradation [24]. Therefore, we included a statistical uncertainty of the material properties in our numerical simulation. Following experimental and numerical studies, we determined a coefficient of variation of 20% for the elastic properties of real bone [25, 26]. For the manufactured materials, such as artificial bone and PMMA bone cement, a coefficient of variation of 10% was assumed [27, 28]. For titanium alloy Ti6Al4V a Young’s modulus of 110000 MPa is reported [29, 30], as also used in other finite element studies [22, 31]. Jebieshia et al. [32] conducted tests on Ti6Al4V alloys from various manufactures and obtained a coefficient of variation of 3.6%.

### Methods for results evaluation

The probabilistic numerical study for the material property uncertainty was implemented using Latin hypercube sampling of these input properties. Using a normal distribution of parameter values [33], a total of 400 samples were taken across ten configurations, with seven for the real bone model and three for the artificial bone model. The probabilistic finite element analyses (FEA) addressed the uncertainty of material properties, which are specified in Table 1 through the mean values and standard deviations (SD) of the Young’s moduli. Material properties were sampled using Latin hypercube sampling and these values were then utilized to generate and run the FEA simulations of each sample point. The probabilistic FEA models were created by generating input files through Python scripts interfaced with the FEA software. This approach allowed for a comprehensive investigation of variability and uncertainty within the biomechanical model, thereby enhancing the reliability of the simulation outcomes.

As bones can experience both ductile and brittle failure [34], we analyzed the stress distribution in the bone using von Mises’ yield criterion and Rankine’s theory (maximum principle). Von Mises’ yield criterion is applicable to ideally ductile materials, while Rankine’s theory describes brittle failure in materials [35]. Since titanium exhibits ductile behavior, we assessed the stresses in the implant using von Mises’ yield criterion. The mean maximum stress and standard deviation were determined for the 400 simulations conducted on the implant, cortical bone, and cancellous bone. Subsequently, stresses at critical regions of the bones were visualized as a function of the angle *φ*. For the talus cancellous bone, the region chosen was the intramedullary nail’s upper rear end. For the talus cortical bone, stresses were analyzed in the region of the top locking screw medially. In the calcaneus, the region for determining stresses in the cortical bone was selected close to the middle screw on the medial side. For the cancellous bone in the calcaneus, the reference region was near the lowest calcaneus screw on the medial side.

Furthermore, the objective was to study the load transfer along the length of the implant. To this end, the torsional, forward, and sideways bending moments resulting from the integration of the normal and shear stresses within the implant were analyzed [36]. Consequently, an implant coordinate system was integrated along the principal axis of the implant. The coordinate system implementation and extraction of the section moment data were again implemented with a Python script for the FEA software.

To be able to distinguish between normal and tangential relative displacements in the subtalar joint gap, we developed a method for identifying the normal and tangential components of the local relative displacements of the bone pair. In this method, detailed in S1 Appendix, we determined the closest distance to the talus over the entire joint surface of the calcaneus and the local normal vector of the joint surface on the talus joint surface. Each relative displacement between two nearest points is then split into a normal component perpendicular to the joint surface of the calcaneus and a tangential component parallel to it. Tangential and normal displacements for the entire joint gap were visualized for neutral, maximum eversion and inversion configurations. For each tangential and normal displacement, a point on the posterior articular talar facet exhibiting the greatest discrepancy between maximum eversion and maximum inversion was selected. The displacement of these points was studied over varying values of the eversion/inversion angle *φ*. These displacements were then compared to the mean section moments within the implant near the joint gap area. To investigate the relationship between the displacements and the section moments, a regression analysis was performed using a Python script. A linear regression function was derived to model the displacements as a function of the section moments. Within this analysis, the coefficient of determination (*R*^2^) for the relationship was calculated to assess the goodness of fit of the regression model.

Additionally, FEA was also performed with the material parameters of the artificial bones (Table 1), to establish a reference data set, for experimental studies using artifical bone specimens.

## Results

In this section the results of the probabilistic nonlinear numerical study are shown. The numerical results include the mechanical stresses in the implant and the bones, the section moments in the implant, and the relative displacements within the joint gap.

### Stress analysis of the intramedullary nail and the cortical/cancellous bone

**Fig 4:**
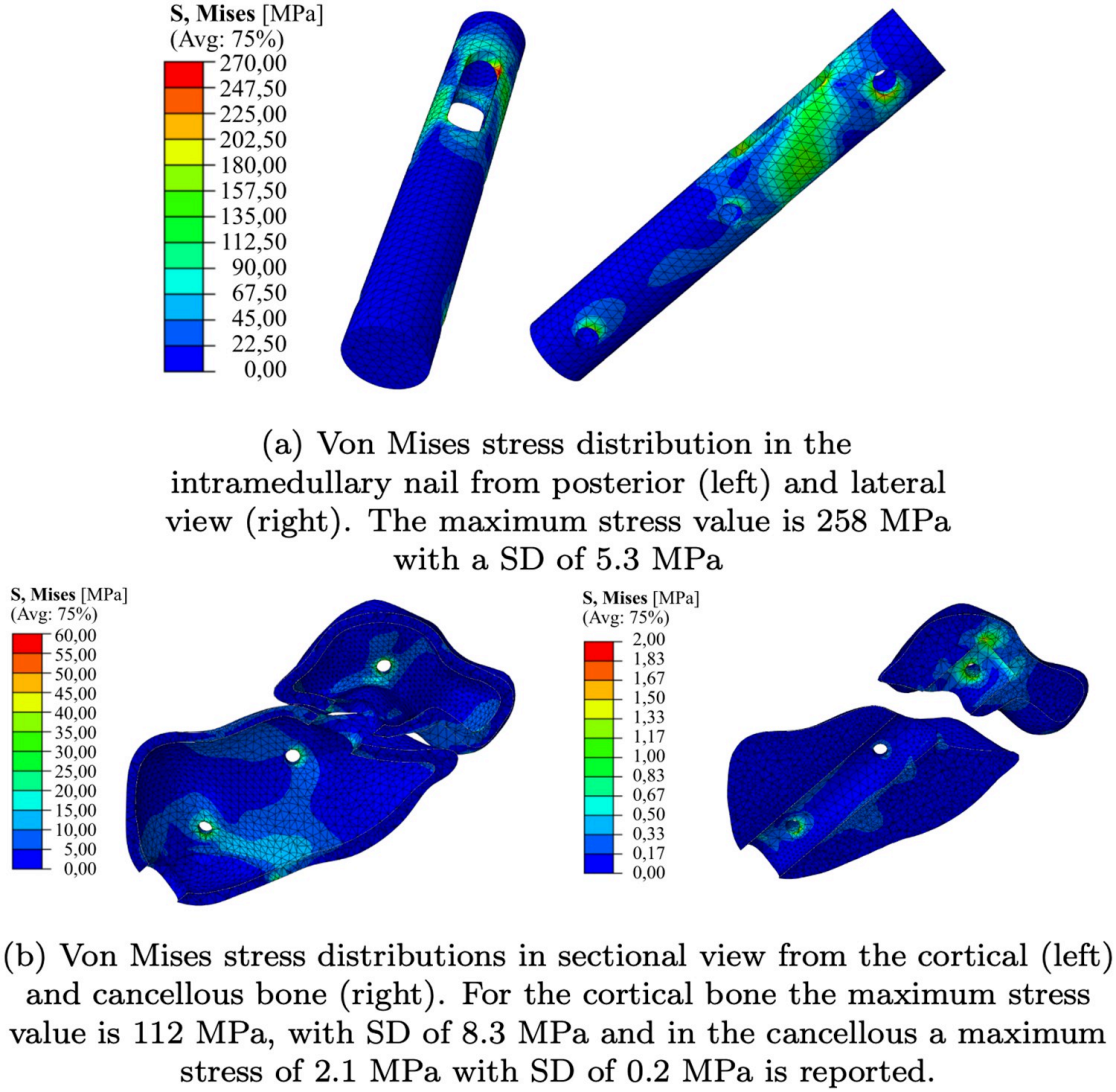
Von Mises stress distributions at maximal inversion. Von Mises stress distributions in the implant, cortical and cancellous bone at the configuration of maximal inversion (*φ* = −20°). The results shown are the mean values of the probabilistic FEA study.

In the maximum inversion configuration of *φ* = −20°, the intramedullary nail experienced the highest level of stress. There, the highest stresses occured in the area near the slotted hole of the Calcanail and in the drill hole area of the locking screws, as illustrated in Fig 4. The maximum stress of about 258 MPa with a SD of 5.3 MPa. This value is approximately 149% of the maximum stress in the neutral configuration and 116% in comparison to the maximum eversion configuration (see Table 2).

**Table 2:** Results of the probabilistic finite element analysis: Maximum stresses in the implant (von Mises criterion for ductile material behavior) and in the bones (von Mises and Rankine criterion to account for both ductile and brittle material behavior). All values are given with their mean value and the standard deviation (SD).

Stress hypothesis	Configuration	Intramedullary nail (mean (SD)) [MPa]	Cortical bone (mean (SD)) [MPa]	Cancellous bone (mean (SD)) [MPa]
von Mises (for ductile materials)	max. inversion	257.5 (5.3)	112.2 (8.3)	1.6 (0.2)
	neutral	172.3 (9.2)	62.5 (7.3)	1.6 (0.2)
	max. eversion	222.2 (3.7)	77.7 (10.5)	2.1 (0.2)
Max. principal (for brittle materials)	max. inversion	-	81.4 (9.6)	1.8 (0.2)
	neutral	-	81.4 (10.3)	1.7 (0.2)
	max. eversion	-	103.2 (13.7)	2.1 (0.3)

The greatest stresses in the bones were typically observed in the regions in contact with the locking screws, the upper end of the intramedullary nail, and the navicular bone. The highest stresses were identified in the eversion and inversion configurations. In a direct quantitative comparison, the inversion configuration exhibited the highest maximum stress on the cortical bone, with a value of 112 MPa and a standard deviation of 8.3 MPa. This maximum stress was 180% and 144% higher than that observed in the neutral and eversion configurations, respectively (see Table 2). The highest stresses in the cancellous bone were observed in the contact zones located at the upper portion of the talus and the locking screws. These stress distribution in cortical and cancellous bone are illustrated in Fig 4 during the maximum inversion configuration. The sagittal plane presents a sectional view.

**Fig 5:**
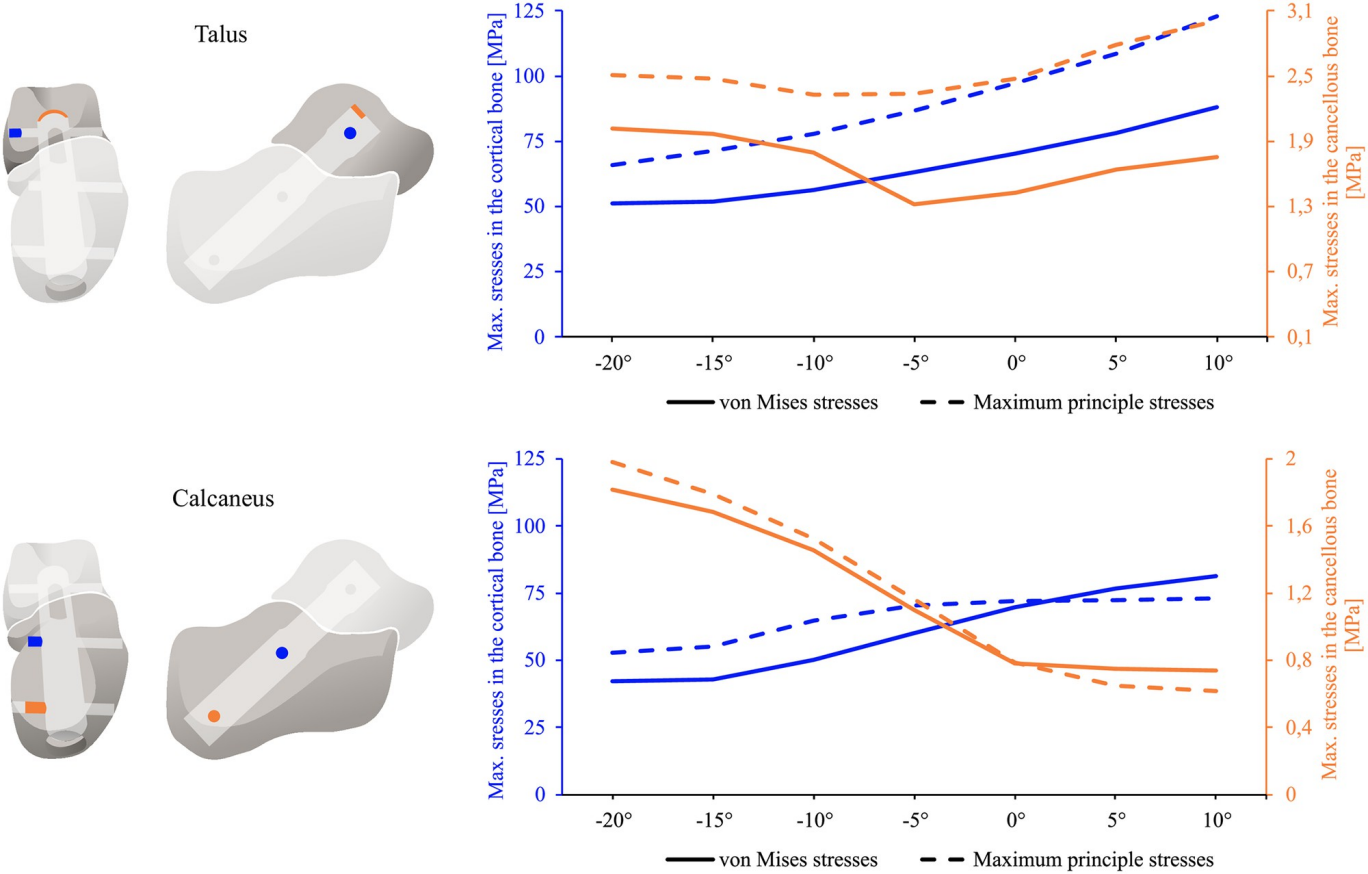
Stress distribution in talus and calcaneus related to inversion/eversion angle. Stresses in talus (top) and calcaneus (bottom) as a function of the inversion/eversion angle. The areas with maximal stresses were chosen for evaluation and are marked blue for cortical bone and orange for cancellous bone.

To gain further insight into the variation of stresses with the angle *φ*, an investigation was conducted into the von Mises and maximum principal stresses at locations of maximum stress. In the talus, the stresses based on the maximum principal hypothesis consistently exceeded those based on the von Mises hypothesis. Notwithstanding the aforementioned discrepancy, both stress curves exhibited a comparable qualitative pattern (see the top of Fig 5). At the upper posterior end of the intramedullary nail (indicated by orange), the cancellous bone exhibited elevated stress levels at both *φ* = -20° and *φ* = 10°. In contrast, the stresses in the cortical bone at the top locking screw, in the medial region (indicated by blue), demonstrated an increase as the angle of inclination increased. However, it is worth noting that at *φ*≤ -10°, the location of the greatest stress in the cortical bone of the calcaneus was on the lateral side of the top locking screw and almost as high as on the medial side at *φ* = 10°.

The calcaneus also demonstrated stress variations as a function of the inversion/eversion angle. The maximum cortical stress near the middle screw on the medial side was obtained during maximum eversion configuration (indicated by blue in the bottom of Fig 5). When investigating other reference areas, such as the region near the screw on the lateral side, it was observed that the cortical bone of the calcaneus experienced maximum stress in the maximum inversion configuration. The highest stress in the cancellous bone was found in the area of the lowest screw also in the maximum inversion configuration (indicated by orange). The comparison of the von Mises and maximum principle stresses revealed that the maximum principle stresses were greater for the different inversion load cases and smaller for the eversion load cases. In the neutral configuration, the von Mises and maximum principle stresses were almost identical. For the selected positions in the calcaneus, the differences between the von Mises and maximum principle stresses were relatively small.

### Section moments in the intramedullary nail

The section moments within the implant provide insight into the load transfer along its principal axis. In the neutral configuration, the moments with the greatest magnitudes occur for the bending moments around the *y*-axis and *z*-axis of the implant coordinate system. The bending moment around the *y*-axis results in a sideways bending, while the bending moment around the *z*-axis corresponds to a bending forward. The global maxima are located at the middle locking screw and in the area between the middle and upper locking screws. The torsional moment around the *x*-axis was relatively low.

In the maximum eversion configuration, the section moment distribution was qualitatively similar to that of the neutral configuration. The forward bending moment around the *z*-axis remained almost constant in magnitude. However, the sideways bending moment around the *y*-axis and the torsional moment were nearly three times larger in magnitude.

**Fig 6:**
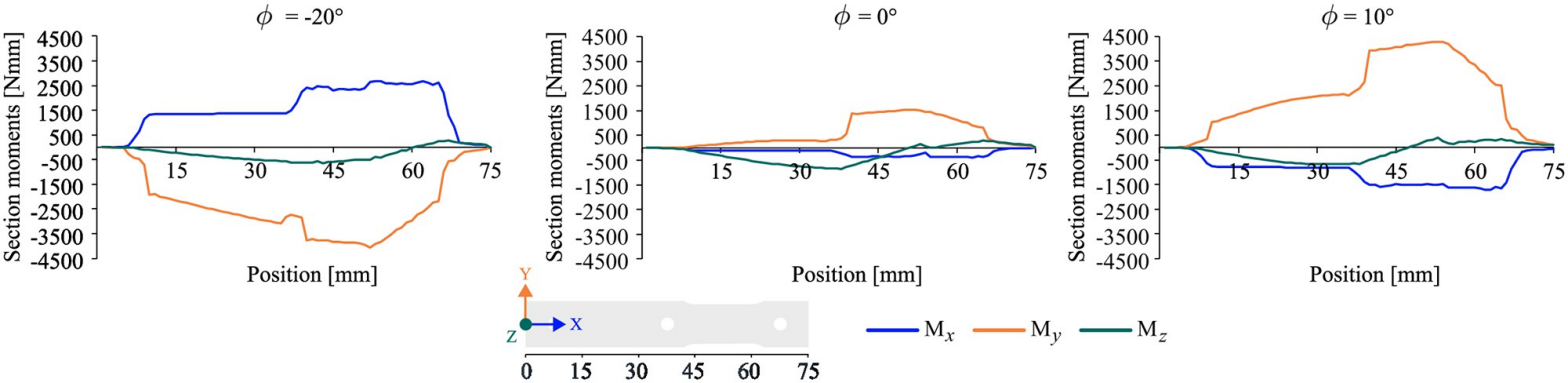
Loading of the calcaneal implant. Section moment curves along the length of the calcaneal implant. The results are shown for the maximal inversion, neutral and maximal eversion configuration. *M*_*x*_ and *M*_*y*_ are bending moments within the calcaneal implant and *M*_*x*_ is acting as a torsional moment.

In comparison to the maximum eversion configuration, the maximum inversion configuration appears almost symmetric. The torsional moment now acted in the opposite direction and was relatively constant over the length with pronounced localized changes in the vicinity of the locking screws. The sideways bending moment around the *y*-axis decreased almost linearly with a minimum in the area of the elongated hole and then increases almost linearly again. The magnitude of the minimum was nearly as high as that observed in the eversion configuration. The torsional, forward, and sideways bending moments along the length of the intramedullary nail is illustrated in Fig 6 for the aforementioned load configurations. In order to gain insight into the impact of section moments in the implant, the subsequent chapter will present a detailed analysis of the relative displacements within the joint gap.

### Relative displacement analysis of the joint gap

**Fig 7:**
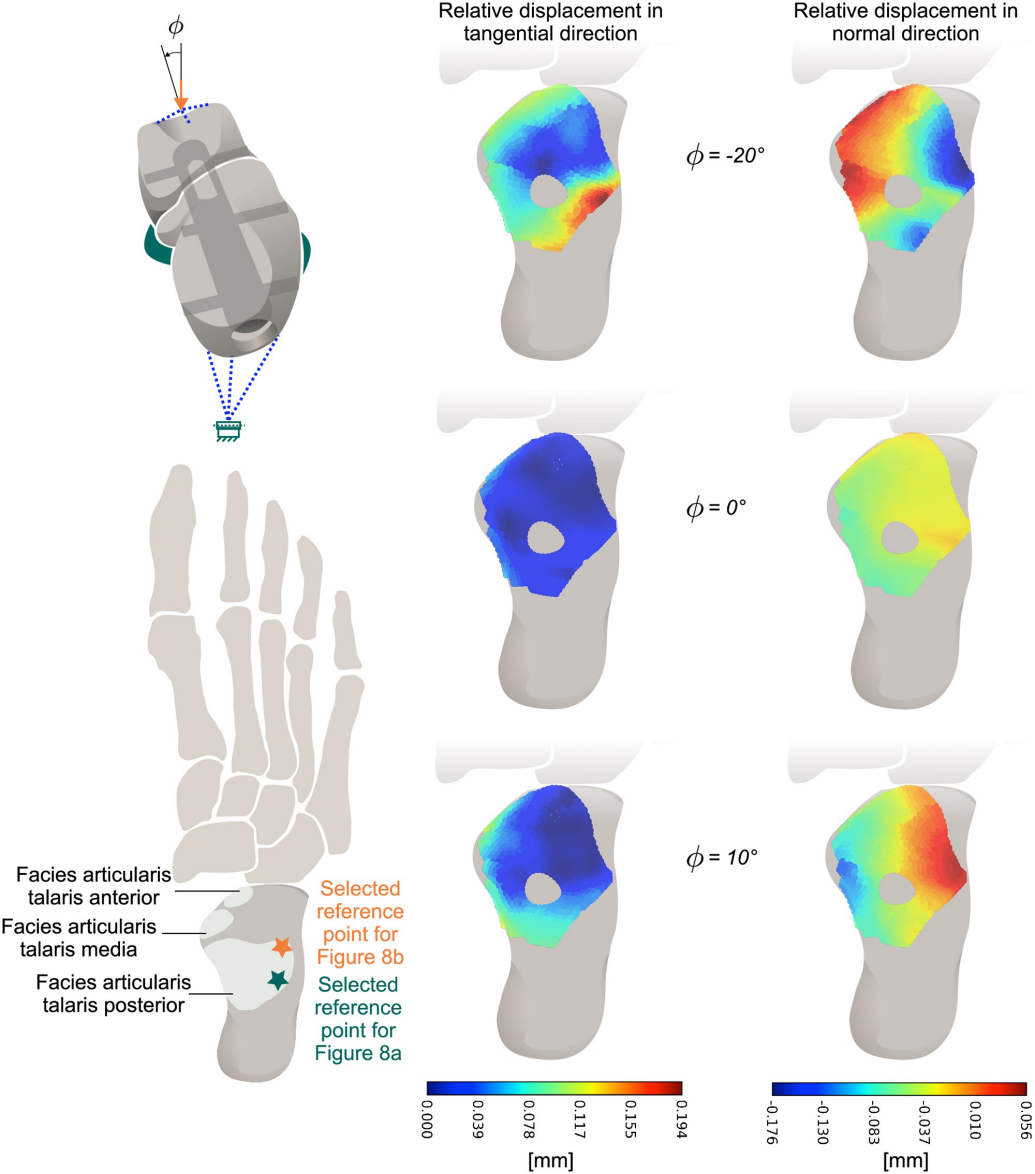
Relative displacement in the joint gap. Relative displacement in tangential (left side) and in normal direction (right side) in the joint gap at the different load configurations.

**Fig 8:**
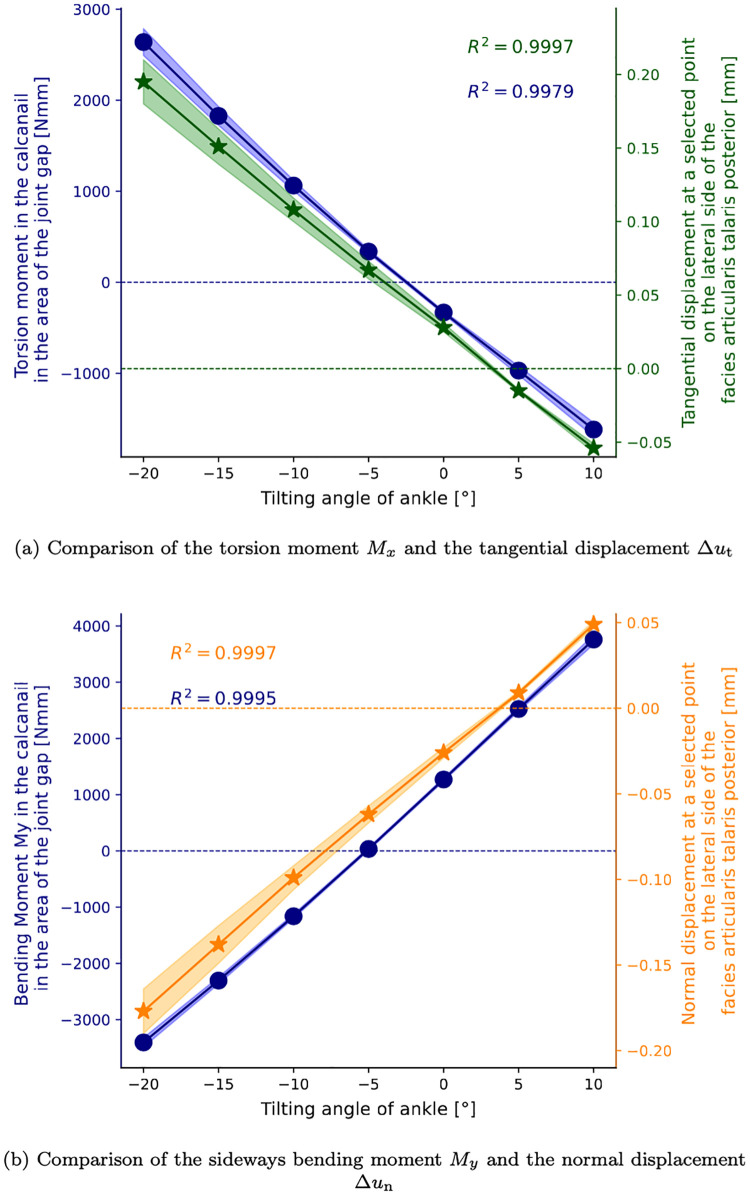
Comparison of section moments and relative displacements. Comparison of section moments in the intramedullary nail and relative displacements at selected points on the lateral side of the posterior articular talar facet (see Fig 7) depending on the angle. The shaded area represents the standard deviation obtained for probabilistic FEA, taking into account the material variance (Table 1).

The relative displacement in the joint gap was distinguished into normal and tangential displacement, as the literature describes disparate effects on bony healing as discussed above. The tangential displacement is observed to be the lowest in the neutral configuration, in comparison to the maximum eversion and maximum inversion configurations. This outcome is attributed to the relatively low torsional moment exerted on the intramedullary nail under the specified load condition. The lower tangential displacements in the neutral configuration are indicated by the data presented in Fig 7, as indicated by the dark blue area on the left side. As the magnitude of the inversion/eversion angle *φ* increases, so does the magnitude of the tangential displacements. A similar effect is observed for the torsional moment in the intramedullary nail in the area of the joint gap (see Fig 8a). The higher the torsional moment within the implant, the higher is the tangential displacement on the lateral side of the posterior articular talar facet (point marked in Fig 7). The regression analysis shows that both the torsional moment and tangential displacement exhibit a linear dependence on the inversion/eversion angle. The linear relationship of the tangential displacement Δ*u*_t_ and the torsional moment *M*_*x*_ was found to be:

$$\begin{array}{c} \Delta u_{\text{t}}\left(M_{x}\right)=5.86\cdot 10^{-5}\;\frac{1}{N}\cdot M_{x}+0.044\;\text{mm}\;\;\left(R^{2}=0.9989\right) \end{array}$$ (1)

Similarly, the relative displacements in the normal direction were found to be lower in the neutral configuration, consistent with the bending moment behavior discussed in the previous section. As illustrated in Fig 7 (on the right-hand side), this expectation is once more clearly demonstrated. In the neutral position, the displacement is reduced. In the area of the anterior and lateral articular talar facets, the relative normal displacement is negative for inversion and positive for eversion. In other words, the typical displacement of this point increases for higher ankle inversion/eversion angle, as illustrated in Fig 8b. This corresponds to the bending moment, which also increases linearly with the inversion/eversion angle. The linear relationship between normal displacement Δ*u*_n_ and sideways bending moment *M*_*y*_ is obtained as:

$$\begin{array}{c} \Delta u_{\text{n}}\left(M_{y}\right)=3.11\cdot 10^{-5}\;\frac{1}{N}\cdot M_{y}-0.067\;\;\text{mm}\;\left(R^{2}=0.9985\right) \end{array}$$ (2)

The probabilistic analysis also allows to identify the effect of the individual material parameters on the mechanical response of the bone/implant system. The Young’s modulus of cortical bone has a great impact on both tangential and normal displacement, compared to the Young’s moduli of cancellous bone and titanium. The Young’s modulus of PMMA bone cement did not have a significant influence on the displacements.

### Comparison with artificial bones

**Fig 9:**
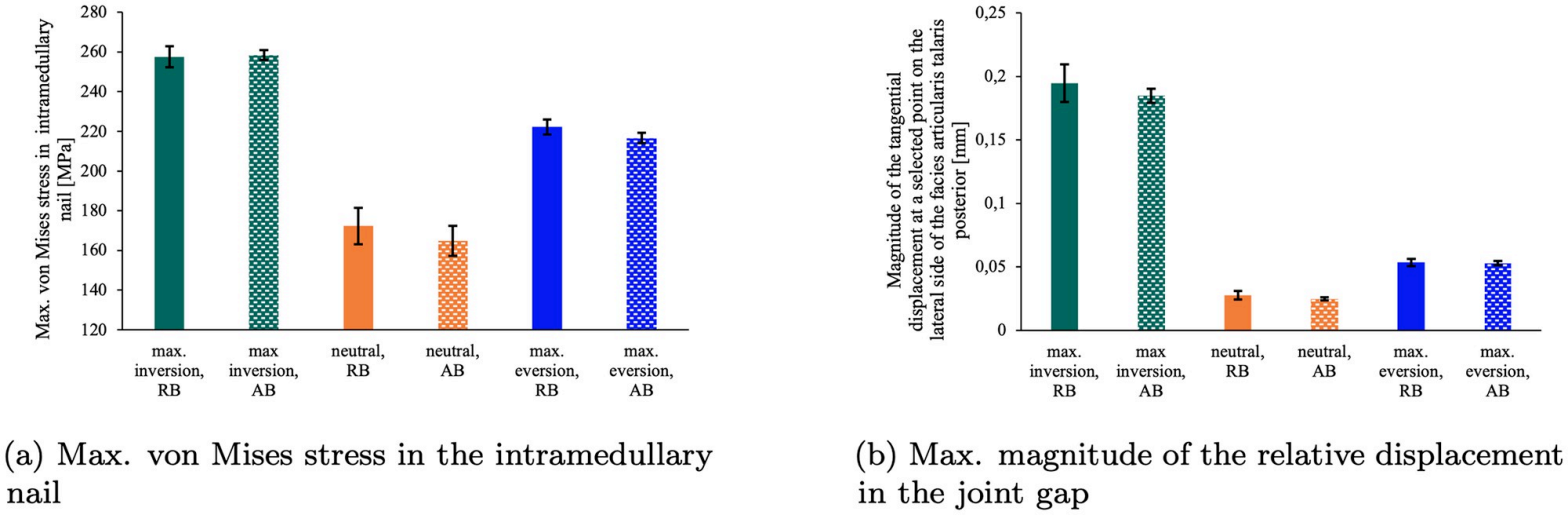
FEA results comparison between real and artificial bone. Comparison of the results of the FEA with real (RB) and artificial bone (AB). Error bars indicate the standard deviation, taking into account the variance in the input material parameters (Table 1).

A comparison of the FEA results with the selected material parameters for real and artificial bone shows that the differences were insignificant in relation to the coefficient of variation for the material parameters. These findings are depicted in Fig 9. The maximum von Mises stresses in the intramedullary nail and the magnitude of the tangential displacement in the joint gap exhibited average differences of 2% and 6%, respectively, between the FEA models with real and artificial bone.

## Discussion

The results of the stress analysis, section moments in the intramedullary nail, and relative displacement analysis of the joint gap can be utilized to elucidate the biomechanical conditions that influence the bony fusion of the subtalar joint. These insights can provide a foundation for further research and clinical studies on subtalar arthrodesis techniques.

The stress results obtained show good agreement with the values reported in the literature. Pang et al. (2014) determined a maximum stress of 285 MPa in titanium osteosynthesis plates with screw fixation, while values around 95 MPa were determined in the cortical bone [41]. These results are in agreement with our stress values. Wong et al. (2016) calculated maximum von Mises stresses in the cancellous bone of 3.21 MPa, and Ni et al. (2019) reported a maximum von Mises stress of 98 MPa in an intramedullary nail for the treatment of calcaneus fractures [22, 37]. Wang et al. (2021) found von Mises stresses in screwed osteosynthesis plates between 106 and 695 MPa [31]. Our study shows similar stress distributions, although it should be noted that the results are not directly comparable due to different loading situations and conditions. The validity of the model is also supported by the comparison of the relative displacement in the joint space. Ni et al. (2019) found a displacement of 0.07 to 0.1 mm in bone fragments stiffened with intramedullary nail, which is consistent with our results [22]. Although the specific conditions differ, the study by Ni et al. addressed a similar mechanical problem, which strengthens the comparability. Another aspect of validation is the comparison between the results for artificial bone and real bone. This included probabilistic FE analyses for artificial bones to verify the sensitivity of the model to changes in material properties. The results were plausible. Overall, the extensive comparisons and analyses confirm the validity of the finite element model.

Maximum eversion and inversion configurations with *φ* = 10° and *φ* = -20° exhibited the highest stresses in the talus and calcaneus. Conversely, the configurations with lower angles yielded the lowest stresses. Therefore, it can be inferred that bone stress was related to the magnitude of angle *φ*. To assess the stresses in both the intramedullary nail and bone, a comparison was made with the literature. Typical titanium alloys used for implants have a tensile strength ranging from 500 to 1100 MPa [42, 43]. The maximum stress obtained was lower than the minimum tensile strength. Therefore, the intramedullary nail was not prone to fail in any configuration. According to the study by Bayraktar et al. on the material properties of the femur, the stress limit of the cortical bone is 107.9 MPa [44]. Therefore, the maximum stresses in the cortical bone were critical in the inversion and eversion configuration. According to studies of the cancellous bone of the calcaneus by Bouxsein et al., the strength of this cancellous bone is 1.49 MPa [39]. Another study by Mittra et al. gave a strength of 1.9 MPa for the cancellous bone of the calcaneus [45]. Thus, the calculated maximum stresses for the cancellous bone might lead to local damage in all configurations under consideration.

The response of the intramedullary nail to the applied load varies significantly across different configurations, with the most significant changes observed in the sideways bending moments around the *y*-axis and in the torsional moments. In the neutral configuration, the forward bending moment around the *z*-axis and the sideways bending moment around the *y*-axis dominated. When everted medially by 10°, the sideways bending moment around the *y*-axis and the torsional moment increased, particularly in the joint gap area. When inverted laterally by -20°, the sideways bending moment around the *y*-axis and the torsional moment change direction due to the altered lever arm of the vertically applied force. At the locations of the locking screws, there were pronounced changes in the moment curves due to the screws being connected to the bone and transfer forces and moments. Fig 6 shows that the intramedullary nail was minimally impacted below the lower locking screw. The highest moments were located in the joint gap area between the middle and upper locking screws. This suggests that the section moments in the intramedullary nail had a significant impact on the relative displacement in the joint gap. The coefficients of determination were nearly 1, indicating linear relationships.

A higher torsional stiffness of the intramedullary nail would decrease the movement in the joint gap that is directly affected by the section moments. This could increase the probability of achieving the desired fusion of the bones, according to several studies reporting different effects of axial and shear movement on the healing of bones. Shear displacements may delay the osseous consolidation. Epari et al. [8] found that large interfragmentary shear movements produce comparable strains but less fluid flow and pressure than moderate axial movements, suggesting that the mechanical stimuli from shear and torsion differ significantly from axial forces. The pivotal role of mechanical signals in modulating biological processes at the fracture site was underscored by Augat et al. [9], who particularly emphasized the harmful impact of shear movement on healing and recommended proper fracture fixation to avoid it. In their numerical models, Steiner et al. [13] demonstrated that axial compression is generally more beneficial for fracture healing than shear movements, with translational shear being particularly detrimental. Furthermore, Epari et al. [46] discussed the importance of mechanical boundary conditions and the interaction between local mechanical environments and tissue differentiation during bone repair, reinforcing the need to minimize shear forces to optimize healing. Furthermore, if the opening and closing movement in the joint gap should be reduced, it would be necessary to decrease the impact of the sideways bending moment in the intramedullary nail around the *y*-axis according to the intramedullary nail coordinate frame. This can be achieved by increasing the respective bending stiffness of the intramedullary nail in the joint gap area.

The study findings suggest that FEA using both real and artificial bone produces comparable results with negligible differences between them. This outcome was expected due to the similar assumed material properties, suggesting that artificial bones could be used in experimental studies.

It is important to acknowledge the limitations of the study. The material properties of bone were assumed to be homogeneous, elastic, and isotropic, which may not accurately represent the complex, heterogeneous nature of cortical and cancellous bone. Additionally, the linear elastic behavior assumption precluded consideration of plastic deformation, which may occur under certain loading conditions and could influence the long-term performance of the implant. Furthermore, the model’s geometry was simplified. The simulation did also not include muscles, tendons, and other soft tissues. However, it has been shown by many researchers that such simplified models can be used to understand fundamental biomechanical relationships [47–49]. Simplified models considering linear elastic behavior are also widely used in form of musculoskeletal full body models that are used to understand effect of joint kinematics, movements and muscle contribution [50–53]. In general, the talus can be subjected to more complex loading conditions than those represented by the simulated single-leg stance with varying inversion/eversion angles of the ankle. Furthermore, the study did not consider the impact of cyclic loading on gait and other activities. Hence, the interaction between the implant and the surrounding bone over time, including potential bone remodeling and the long-term fatigue strength of the implant, was not taken into account. This is also a typical assumption of basic biomechanical studies, which lay the foundation for more complex numerical studies to determine the effect of these factors.

The present non-linear FE model has proven to be comparable to experimentally validated studies and provides valuable insights into the fundamental principles of locking nails used for fixation and promotion of successful subtalar joint arthrodesis. The probabilistic FEA facilitated an examination of the uncertainty associated with material input parameters, thereby enhancing the comprehensiveness of the biomechanical understanding.

## Conclusion

The probabilistic finite element analysis (FEA) employed in this research accounts for variations in mechanical conditions and material properties, thereby enhancing the reliability of the findings. This study highlights the considerable influence of foot eversion and inversion on the healing process during the initial postoperative phase, due to the presence of critical stress concentrations in the bones and augmented displacements in the joint gap. The methodology allows for separation of normal and tangential displacements in the articular gap, providing valuable insights into relative displacements. This is of special importance as the role of tangential displacements is intensively discussed in literature and various studies have demonstrated that such displacements may delay osseous consolidation [8, 9, 14]. The study thus recommends that excessive inversion or eversion of the hindfoot be avoided or limited during the critical initial postoperative phase, and that a lower leg orthosis be used to facilitate patient mobilization. Overall, this research emphasizes the importance of understanding biomechanical healing conditions, reinforcing the need for ongoing studies in this field to enhance clinical outcomes in bone healing.

## Supporting information

S1 Appendix Calculation of relative joint gap displacement. (PDF)

S1 File Results of the FEA for real and artificial bone. (ZIP)
