# Supplementary material for: Biomechanical conditions of subtalar joint arthrodesis with calcaneal locking nail: A probabilistic numerical study
Source: PLoS One. 2024 Nov 20;19(11):e0314034. doi: 10.1371/journal.pone.0314034 (PMC11578502; doi:10.1371/journal.pone.0314034)
Supplement: S1 Appendix — (PDF) [file pone.0314034.s001.pdf]

S1 Appendix

Calculation of relative joint gap displacement

To distinguish between normal and tangential relative displacements in the subtalar joint gap, a local normal vector is determined for each point on the articular surface of the calcaneus. Since a mesh node in an FE-model does not have a normal, it must be interpolated from the normals of the surrounding element faces. The areas of the faces determine the weighting. First, for each node on the articular surface of the calcaneus, all adjacent element faces are determined. Then, for each triangular element face, an outward normal $\vec{n}_{\mathrm{f}}$ can be calculated.

To calculate the influence of face normals on the node normal, we need to determine the areas of the faces from their corner coordinates. Then, we can calculate the node normal by summing the weighted adjacent element face normals and normalizing the result:

$$\vec{n}_{\mathrm{n}} = \sum^{e} \vec{n}_{\mathrm{f}} A_{\mathrm{f}}. \tag{1}$$

To obtain local relative displacements, each node on the articular surface of the calcaneus is paired with the nearest node on the surface of the talus. In a simulation both the calcaneal and the paired talus node will be subject to three-dimensional displacements $\vec{u}_{\mathrm{c}}$ and $\vec{u}_{\mathrm{t}}$. The relative displacement between them is

$$\Delta\vec{u} = \vec{u}_{\mathrm{c}} - \vec{u}_{\mathrm{t}}. \tag{2}$$

To determine which fractions of this relative displacement are locally normal to the calcaneal articular surface the angle θ between the node normals and corresponding vectors of relative displacement is calculated:

$$\theta = \cos(\vec{u}, \vec{n}). \tag{3}$$

The normal and tangential relative displacements Δu_{rn} and Δu_{rt} can then be determined as

$$\Delta u_{\mathrm{n}} = \cos\theta |\Delta\vec{u}|, \tag{4}$$

$$\Delta u_{\mathrm{t}} = \sin\theta |\Delta\vec{u}|. \tag{5}$$
